# Single Cell Transcriptomics to Understand HSC Heterogeneity and Its Evolution upon Aging

**DOI:** 10.3390/cells11193125

**Published:** 2022-10-04

**Authors:** Léonard Hérault, Mathilde Poplineau, Elisabeth Remy, Estelle Duprez

**Affiliations:** 1 I2M, CNRS, Aix Marseille University, 13009 Marseille, France; 2 Epigenetic Factors in Normal and Malignant Hematopoiesis Lab., CRCM, CNRS, INSERM, Institut Paoli Calmettes, Aix Marseille University, 13009 Marseille, France; 3 Equipe Labellisée Ligue Nationale Contre le Cancer, 75013 Paris, France

**Keywords:** HSC aging, single cell transcriptomic, bioinformatics, Boolean modeling

## Abstract

Single-cell transcriptomic technologies enable the uncovering and characterization of cellular heterogeneity and pave the way for studies aiming at understanding the origin and consequences of it. The hematopoietic system is in essence a very well adapted model system to benefit from this technological advance because it is characterized by different cellular states. Each cellular state, and its interconnection, may be defined by a specific location in the global transcriptional landscape sustained by a complex regulatory network. This transcriptomic signature is not fixed and evolved over time to give rise to less efficient hematopoietic stem cells (HSC), leading to a well-documented hematopoietic aging. Here, we review the advance of single-cell transcriptomic approaches for the understanding of HSC heterogeneity to grasp HSC deregulations upon aging. We also discuss the new bioinformatics tools developed for the analysis of the resulting large and complex datasets. Finally, since hematopoiesis is driven by fine-tuned and complex networks that must be interconnected to each other, we highlight how mathematical modeling is beneficial for doing such interconnection between multilayered information and to predict how HSC behave while aging.

## 1. Introduction: Problematic of HSC Aging

Aging leads to a decline in the functions of the hematopoietic and immune system, which in the elderly results in an increased risk of infection, poor vaccination efficacy, anemia and blood cancers [1]. It is now well established that age-related dysfunction of the entire hematopoietic system originates from hematopoietic stem cells (HSCs), which lose their fitness over time. HSCs at the top of the hematopoietic hierarchy reside in the bone marrow (BM) and are able to maintain the system thanks to their capacity of self-renewal and multipotent differentiation, which allows them to produce all blood cells throughout the lifetime of an individual. Over time, HSCs progressively lose their regenerative capacity and show an attenuated lymphoid potential, counterbalanced by an increased myeloid potential [2,3,4], which may contribute to the reduction in adaptive immune cells and immunosenescence in older individuals. Throughout the years, it has become clear that the HSC compartment is highly heterogeneous, with phenotypically identical but genetically different cells and that clonal evolution of HSC with different potential is intimately linked to aging [5]. This is supported by age-related clonal hematopoiesis (ARCH); the clonal expansion of HSCs carrying specific, disruptive, and recurrent genetic variants, which disproportionally contribute to blood cell production [6]. This clonal hematopoiesis is associated with hematological pathologies, such as myelodysplastic syndromes, acute myeloid leukemia and chronic lymphocytic leukemia [7,8,9] as well as with cardiovascular diseases [10].

**Figure 1:**
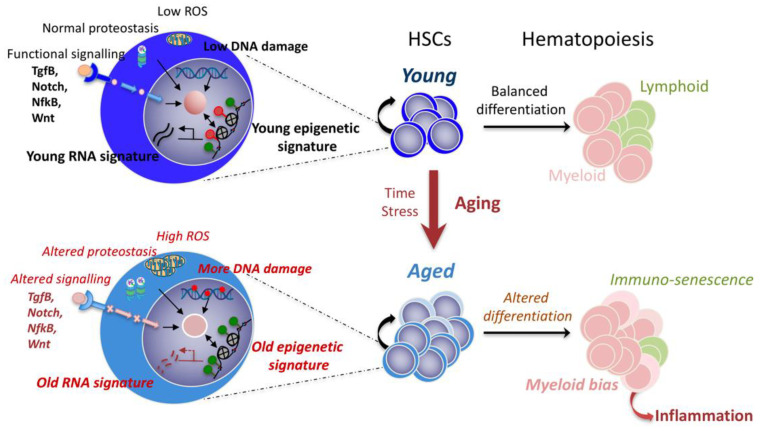
Cellular and molecular changes of aged HSCs. On the left, the zoom into young (**top**) and old (**bottom**) HSCs summarizes four biological processes that have been proposed to be involved in HSC aging: metabolism (ROS, proteostasis), DNA damage, epigenetics (green and red circles: active and repressive histone marks) and signaling. Interconnections between these processes lead to an altered function revealed by changes in transcriptome signatures. On the right, hematopoiesis is schematized. Young hematopoiesis is characterized by a balanced differentiation, leading to accurate levels of myeloid and lymphoid cells. With aging, intrinsic changes in HSCs occur, resulting in a myeloid bias and immunosenescence.

It is, therefore, legitimate that experimental efforts in recent years have focused on understanding the intrinsic deregulations of aged HSCs. At present, much of our knowledge comes from genetically modified mouse models and comparative transcriptomic studies of young and old mouse HSCs. Although the data obtained are often divergent between different laboratories, a large number of distinct intrinsic molecular alterations associated with aging have been identified. A consensual picture indicates that aged HSCs have aberrant regulation of genes involved in myeloid and lymphoid differentiation [11], an increase in megakaryocyte–platelet markers [12,13], a pro-inflammatory signature [14] and cell cycle gene deregulation [15]. These aged signatures are associated with an increase in DNA damage, and with changes in metabolism and mitochondrial homeostasis, in chromatin remodeling (epigenetics), in cell polarity and in cell cycle [4,16,17] (summarized in Figure 1).

All these changes probably play a role in age-related HSC deterioration, being interconnected and regulated in response to microenvironment signals, such as Tgf-Β, Notch, NF-κB and Wnt [18]. Yet, the direct causal relationship of these changes in HSC aging is still poorly documented. One example that illustrates our lack of understanding is the changes in epigenetic and chromatin organization associated with HSC aging [8,19,20,21]. This epigenetic drift has been observed in different models of aging and at different levels of the chromatin landscape [22] and result in a more open state of the chromatin [23], however, its role in age-related functional alterations of HSCs, including clonal selection, is still not entirely clear. Attempts to link this phenomenon to somatic mutations in the epigenetic machinery found during clonal hematopoiesis [24,25] have not been successful due to the lack of explicit mechanisms, linking genotype to observed phenotype [26,27,28].

## 2. Evolution of Single Cell Transcriptomic Studies: From Quantitative PCR (qPCR) to Single-Cell (scRNA-Seq)

**Figure 2:**
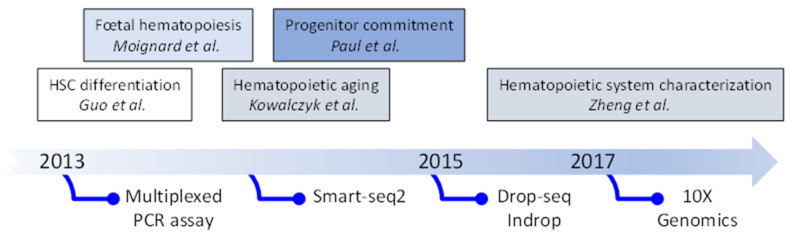
Evolution of single-cell transcriptomic technologies and their application.

Historically, the advance in our knowledge of HSCs has been driven by fluorescence-activated cell sorting (FACS) approaches that allow the purification of a cell population based on the expression of a panel of cell surface markers, whose differentiation potential can be assessed by a functional colony assay in vitro or after transplantation into irradiated mice [1]. This cell-surface marker-based HSC characterization has shaped the classical but largely revisited hematopoietic model, in which the long-term HSC (LTHSC), at the top of the hierarchy, undergoes a lineage commitment through a series of discrete intermediate progenitor stages in a stepwise manner. This approach has helped to categorize short-term HSC (STHSC) and multipotent progenitor populations (MPP2, MPP3 and MPP4) [29,30,31]. However, the differentiation hierarchy has become increasingly complex with a growing number of characterized progenitor subtypes that went hand in hand with the technological evolution of FACS, allowing the simultaneous study of a large number of surface markers. This escalation in the amount of surface markers used led to environmental stress during capture and subsequent functional testing that introduced a laboratory-dependent bias in the way HSCs properties were understood [32]. In the hope of obtaining a complete and unbiased view of the HSC and progenitor compartment, new approaches using transcriptomics at the single-cell level have emerged. The first transcriptomic studies of hematopoiesis at the single cell level were conducted using real-time PCR on hundreds of murine hematopoietic precursors to measure the expression of several dozen intracellular markers, including transcription factors (TFs), known to regulate hematopoiesis [33,34]. These early studies not only provided essential information about the HSC heterogeneity and commitment, but also importantly evidenced the power of single-cell transcriptomics, paving the way for fully unbiased analyses of single cell whole transcriptomes. Concurrently, scRNA-seq technologies evolved to make the sequencing of several thousand cells accessible with integrated fluidic circuits and liquid handling robotics [35]. This has enabled a pioneering study of 2700 murine progenitors, demonstrating their early commitment to distinct lineages well before the common myeloid progenitor (CMP) state [36], as well as a scRNA-seq analysis of early hematopoietic aging characterizing 1100 HSCs and progenitors from young and aged mice [37]. Progressively, scRNA-seq shifted from plate-based technologies, targeting a high sequencing coverage of few cells such as Smart-seq2 platform [38], to droplet-based technologies, such as Drop-seq [39], Indrop [40] or 10X Genomics [41], scaling up the number of characterized cells to tens of thousands, enabling the discovery of new rare cell types. These approaches, although limiting sequencing to the 3′ (or 5′) end of genes and detecting only about 2000–3000 genes per cell, have generated great interest in the HSC community in search of rare cell populations (see below). Figure 2 is summarizing the principal steps. The rapid development of bioinformatics tools and their open access has helped move single-cell transcriptomics from an application in highly specialized laboratories to a widely used technology to apply to HSC-related questions.

## 3. Workflow for Studying Hematopoiesis in Mice

**Figure 3:**
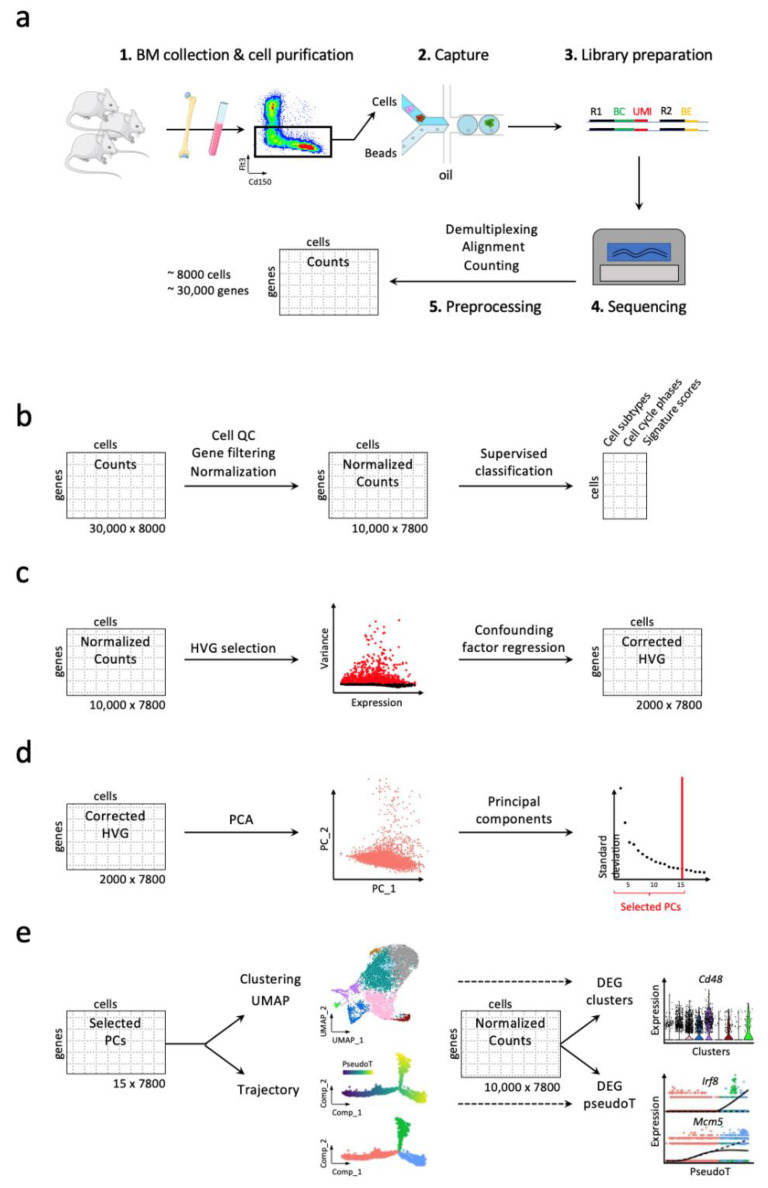
scRNA-seq workflow for studying hematopoiesis in mice. (**a**) Isolation, sorting, capture of cells of interest and preparation of libraries for a pool of mice. Example of a droplet-based technology, R1: Read 1 biological, BC: cell barcode, BE: sample barcode. Primary data processing after sequencing: Demultiplexing of binary base call (BCL) files in FASTQ files that are aligned to the reference genome, then transcript counts per cell are quantified using the unique molecular identifiers (UMIs). In this example, the expression of 30,000 genes is detected for 8000 cells. (**b**) Quality control (QC) of the cells and filtering of lowly expressed genes. In this example 10,000 genes expressed in 7800 cells are conserved. Normalization of counts and some supervised analyses (cell cycle scoring/phase assignment, supervised cell type annotation) can be performed. (**c**) Highly variable genes (HVGs) are selected for dimension reduction and cell clustering. Confounding factors (cell cycle, percentage of mitochondrial transcripts, etc.) can be regressed out during the scaling of the HVG expression. (**d**) A first linear dimension reduction with a PCA to summarize the information. The most informative principal components (PCs) are kept regarding the drop in the percentage of explained variance. (**e**) A clustering and a visualization with UMAP (or tSNE) are conducted on these PCs. In addition, a pseudo-trajectory can be inferred with the selected top PCs. Finally, differentially expressed gene (DEG) analyses between clusters/conditions or along pseudotime (pseudoT) are usually performed on normalized expression data.

The spread of scRNA-seq technologies, which generates high-dimensional sparse matrixes (tens of thousands of expressed genes measured in tens of thousands of cells), has been facilitated by the concomitant development of bioinformatic tools (for review see [42]. Indeed, in order to conduct the analysis of the increasing amount of data, hundreds of bioinformatic tools have been developed and benchmarked, addressing each step of the analysis [43], such as Seurat [44,45,46] or Scanpy, more suitable for very large datasets [47]. These central methodological developments have led to an analysis strategy that is widely used today and whose steps are described in Figure 3. Nonetheless, some biases in the scRNA-seq analysis blur our perception of the heterogeneity of the cell population studied. Such biases are inherent to the sparsity of the data with many so-called dropout events (i.e., when an expressed gene is not detected due to low sequencing coverage) and due to the complexity and number of steps implemented for the scRNA-seq analysis.

### 3.1. Handling High Dimensionality of Raw scRNA-Seq Data

Raw count matrix of scRNA-seq can contain dozens of poor-quality cells and thousands of genes expressed in very few cells or not detected at all that are filtered out in a first preprocessing step (Figure 3b). Then, normalization is applied on the resulting matrix to correct for the varying sequencing depth between the cells. After this step, it can be interesting to conduct supervised cell classification using labelled scRNA-seq dataset previously published to annotate or score cells for confounding factors, such as cell cycle or cell subtypes of interest. Typically, the majority of scRNA-seq studies consists of unsupervised analyses (dimension reduction and cell clustering) that is performed on the normalized and scaled expression of highly variable genes (HVGs) [44,46]. It is worth noting that technical (sequencing depth) or biological (cell cycle score, percentage of mitochondrial transcripts expressed) confounding factors can be corrected when scaling HVG expression (Figure 3c).

Unlike the analysis of bulk RNA-seq data, classically used to characterize transcriptomic differences between two biological conditions (differential gene expression, splicing), the analysis of scRNA-seq data is mainly oriented towards the characterization of cell population heterogeneity (Figure 3d). This heterogeneity is captured by visualizing the cells in a reduced two- or three-dimensional space with dimension reduction techniques, such as tSNE or UMAP, which are often used after an initial linear reduction in dimensions with principal component analysis to summarize the data [48]. Although significant progress has been made with these two-step dimension reduction techniques, a loss of information persists, making biological interpretations conducted in the reduced final spaces difficult. The choice of the dimensionality of the data remains one of the main sources of bias because it is often done heuristically by seeking a decrease in the percentage of variance explained by the principal components (Figure 3d).

### 3.2. Discrete and Continuous Analyses of the Cell Heterogeneity and Gene Marker Identification

Concomitant to UMAP visualization, cell-to-cell transcriptomic variations are typically assessed by detecting clusters of cells in the principal component analysis (PCA) space, which assumes biologically disconnected groups of cells. For continuous processes, such as hematopoiesis, another approach is to consider each cell as a snapshot at a given time point of the process under study, which must be reorganized in a pseudotime along a trajectory from an initial state to one or more terminal or differentiated states. Numerous pseudo-trajectory inference methods have been developed for this purpose [49,50,51,52] whose benchmark highlighted substantial variability in the results. This led to great caution in considering the calculated pseudotime values and the identified branching points [53]. In practice, scRNA-seq data studies of differentiation processes, such as hematopoiesis, now often conduct both discrete and continuous representations of the cells and combine the results to interpret the data [54,55,56].

The identification of markers is the last crucial step of a scRNA-seq analysis, it allows one to draw biological conclusions from the studied model but paradoxically requires a biological expertise of the latter. Marker genes are determined on the basis of their differential expression between different clusters or branches of the pseudo-trajectory (Figure 3e) and will allow the biological characterization of different subsets of cells or states (e.g., proliferation, quiescence for example). Unfortunately, this step is intrinsically impacted by the dropout noise. To overcome this bias, several solutions have been developed, including the use of missing count imputation methods [57,58] and the use of TF activity signatures issued from bulk transcriptomic and epigenomic analyses [59,60]. Both methods have their advantages and disadvantages. The imputation strategy provides a more accurate and unsupervised correction for marker identification but can sometimes overcorrect the data [61], while the use of TF activity signatures effectively mitigates dropout noise, as they typically contain dozens of genes but only allow for analysis of already characterized biological processes. Merging highly similar and possibly redundant cells into metacells [62,63] is an emerging alternative that has the advantage of reducing not only the background noise but also the data size, which is becoming increasingly problematic as the number and size of single-cell datasets increase [64].

### 3.3. Addressing Batch Effect

A particular feature of scRNA-seq studies is their sensitivity to batch effects, often observed in mouse HSC studies due to the need for multiple pools of mice in order to collect enough cells of interest. They are manifested by different sequencing depths between each batch but also by more or less strong activations of genes linked to cellular stress, such as ribosomal or mitochondrial genes. These biases can be avoided or at least strongly mitigated by using cellular indexing of transcriptomes and epitopes by sequencing (CITE-seq) that allows cells from distinct samples to be uniquely labeled. Sequencing of these labels allows each cell to be associated with its original sample [65]. If this is not sufficient, there are effective methods to correct gene expression on a batch basis with linear [66] or more complex [67] models. Batch effect corrections may become problematic when comparison of different experimental conditions (gene disruption, treatment, kinetics) is part of the experimental design. It will then be necessary to juggle with the differences due to experimental conditions and batch effects by integrating the different datasets together in a common space. For this purpose, several methods have been proposed based on the identification of cell state subsets shared between the different datasets, benchmarked in [68]. Data integration is becoming increasingly important, as it is essential for joint analysis of different omics datasets at the single cell scale [46,69,70].

## 4. Understanding HSC Heterogeneity to Grasp Aged HSC Deregulations

The very large number of scRNA-seq studies carried out over the last ten years has greatly increased our knowledge of different biological processes, particularly in the context of hematopoiesis. The interest of laboratories working on HSCs for scRNA-seq approaches is explained by the need to understand, at the molecular level, the heterogeneity of HSCs and its impact on their progeny. It is now admitted that each individual HSC, although sharing the same cell surface marker combination, differs in terms of functional outputs and molecular signatures [71,72]. The numerous recent scRNA-seq studies, which have been enriched with the increase in the number of cells sequenced, have challenged the traditional, hierarchical view of hematopoiesis and have led to the reconsideration of blood cell relationships and the routes by which lineage differentiation occurs [73]. It became evident that hematopoiesis is a continuous differentiation model with a lack of clear delineation between the different subtypes of murine HSCs [54,74] and with the identification of an early lineage priming in subsets of HSCs [55,75]. The continuum of differentiation in the HSC population has also been demonstrated in humans, where the transcriptomes of the HSC and MPP (CD34+ cells) compartment were found to be very similar, suggesting a cloud of HSCs differentiating directly into unipotent progenitors [76,77]. This model has been related to Waddington’s landscapes of cell differentiation in which the differentiation of a cell is represented by a bead going down a hill into diverging valleys, each of which ultimately leads to a different cell type [78,79].

The value of using scRNA-seq to identify and characterize rare populations has been demonstrated in studies seeking to understand the cellular and mechanistic evolution of HSC during aging. The transcriptomic analyses at the single cell level have particularly been useful to clarify the origin of the myeloid bias found in aged HSCs. Although a first study demonstrated a cell autonomous impaired lymphoid differentiation potential of aged MPP4 pinpointing the cellular compartment responsible for lymphoid cell loss with aging [80], most of the following studies suggested a gain in myeloid differentiation potential of aged HSCs. This gain was mainly due to an expansion of identified platelet-primed or megakaryocyte-primed HSCs [55,81]. In line with the increase in myeloid potential observed in old HSCs, several studies demonstrated an expansion with age of HSCs primed to respond to an inflammatory stimulus. Three studies highlighted a clear subpopulation of HSCs primed for interferon stimulus that were prepared to strongly respond to future stress or injuries that are expanded with aging [55,82,83]. In addition, a new group of MPPs have been described [84], which ultimately resemble from a transcriptomic point of view the group of interferon-primed progenitors described in [55,83]. This specific age-related amplification of LTHSCs with mis-regulated interferon signaling is consistent with the concept of inflammaging [85]. Another interesting HSC group, amplified during aging, is the cluster of LTHSCs presenting a TGF signature [55] that may correspond to the accumulation of the HSC subtypes with differential responses to the TGF that was previously identified [86].

The accumulation of non-functional HSCs during aging has also been extensively studied and is thought to be related to the history of HSC cycling activity and an imbalance between self-renewal and the initiation of HSC differentiation [87]. Concerning the regulation of the balance between population maintenance and differentiation, a study based on scRNA-seq suggested an increase in self-renewal, which would be linked to a shortening of the G1 phase of the cell cycle [37]. However, another study showed that the accumulation of non-functional aged HSCs, seemed to originate rather from a blockage of the differentiation of quiescent-aged HSCs biased towards the myeloid lineage [55]. The latter study also showed an increase in the TGF signature in these cells, suggesting a role for TGF signaling in the accumulation of aged HSCs. In parallel, another study explained the accumulation of the aged HSC population by the activation of the JAK-STAT pathway and p53 [88]. Interestingly, these two studies agreed on the markers of this aged HSC population that shared the same key genes (*Hes1*, *KLF* factors, *JunB*, *Nr4a1*, *Cdkn1a*). *EGR1* was found to be upregulated in aged human hematopoietic stem cells, independent of cell cycle phase progression, but was associated with loss of CDK6 and CCND2 during S phase, which would disrupt HSC cell cycle [89]. These studies converge to show an accumulation of cells with several quiescent markers, such as Nr4a1, Junb, and Cdkn1a, which are compatible with observations on the quiescent state of HSCs [90,91]. They are also consistent with the distinction between quiescent and active HSCs and with the role of retinoic acid signaling in maintaining the hypoxic dormant state [92,93]. In addition, a study that implies an important role for Cdc42 activity/polarity in HSCs for driving the symmetric/asymmetric mode of division revealed that the frequency of polar HSCs decreases upon aging, which results in more symmetric divisions with daughter stem cells, with impaired potential [94].

It is important to note that all of the studies discussed above only partially overlap, with some features of aging not replicated across studies. One source of discrepancy may lie in how and when they handle cell cycle bias in their analysis. Indeed, the cell cycle induces variations in the whole transcriptome, confounding signals of interest, such as cell differentiation [95]. Thus, young and aged HSC/MPP heterogeneity in term of priming for specific lineage seems to be better resolved with a regression of cell-cycle effect before dimension reduction [55] than without [37,82]. The partial overlap between studies could also very well be a consequence of heterogeneous aging capture, consistent with the theory of clonal hematopoiesis, which shows that competitive clones emerge and amplify at the expense of others during an individual’s lifetime [96]. One way forward would be the construction of a large single-cell atlas collecting hundreds or even millions of thousands or even millions of cells to provide an in-depth phenotypic description of biological tissues across a wide range of pathological conditions, similar to the human cell atlas [97] or the tumor infiltrating lymphocytes atlas using samples from hundreds of donors [98].

## 5. Coupling the Transcriptome to the Cell Fate

An important issue is to capture the temporal dynamics of the cell population by evaluating the future state of an individual cell. One strategy is to use RNA velocity analysis, which is based on calculating the time derivative of the gene expression state by distinguishing newly transcribed (spliced) mRNAs from mature (unspliced) mRNAs by the presence of introns. The combination of velocities between genes is then extrapolated to estimate the future state of each cell in transcriptome space [99,100]. Although promising, this bioinformatics approach seems poorly suited to the study of hematopoietic cells in which changes in mRNA processing and stability may be key factors in HSC activation [101]. This is observed experimentally by a boost in RNA transcription that induces unexpected and probably erroneous projections of future cell states due to biased velocity estimates [102].

To address the challenge of correctly inferring the cell differentiation trajectory, the best strategy seems to combine scRNA-seq with other single-cell omics technologies and/or lineage tracing approaches [103].

Lineage tracing methods have proven effective in understanding the heterogeneity of the initial HSC population, as well as the clonal relationships between individual HSCs and their progeny [103]. These methods are based on a library of DNA barcodes expressed within a transgene that are stably integrated into the genomes of the HSCs under study. The offspring after multiple divisions of a particular HSC, thus, inherits its own barcode, allowing the assessment of transcriptional changes and functional potential of each cell in the same experiment. In this way, it is possible to trace the progeny of a HSC in an inferred pseudo differentiation trajectory and present an unbiased view of differentiation. Altogether, lineage tracing studies interestingly showed that rather few mature cells had integrated barcodes at the HSC level (apart from the megakaryocytic lineage), suggesting that MPPs rather than HSCs were the main contributors to undisturbed hematopoiesis (reviewed in [104]). When coupled with scRNA-seq, they appeared to be the right way to follow the fate of a given HSC in relation to its transcriptomic signature and have deepened our understanding of HSC differentiation potential. They confirmed the early priming of HSCs to different lineages under physiological conditions [75,105]. They additionally showed, using computational methods of dynamic inference that fate choice occurs earlier than detected by the algorithms and that cells progress smoothly in the differentiation with precise and consistent dynamics [105]. These results were later strengthened by the addition of CRISPR-seq to lineage tracing and scRNA-seq, which clarified that the HSC subpopulation with high self-renewal potential contributed very little to hematopoiesis [106]. Single-cell epigenomic approaches could also be useful in determining the fate of an HSC. This hypothesis is supported by a HSC lineage tracing study, combined with extensive transcriptomic and epigenomic analyses, which demonstrated that epigenetic features, in contrast to the transcriptome, are consistently correlated with the cell fate [107]. Altogether, these approaches reveal the limits of using scRNA-seq alone to distinguish functionally heterogeneous HSC states and emphasized that transcriptionally similar cells can have cell-autonomous bias toward different fate choices [104].

## 6. Network-Based Dynamic Modeling: A Successful Approach to Decipher Hematopoiesis

As discussed above, the HSC fate is driven by complex interaction networks involving signaling, transcriptional and also epigenetic regulations. To decipher such large and complex biological networks and to cope with and take advantage of multiple layers of information, explanatory and predictive mathematical models are beneficial.

Because of the ease of collecting blood or bone marrow samples, which facilitated data generation, the hematopoietic system was early on the subject of mathematical modeling studies [108]. Initially, studies at the level of the production of the different blood cell populations and their interactions were modeled using differential equations [109,110,111]. However, this mathematical formalism requires precise parameters, such as reaction constants or other initial conditions that are important because the behavior of the system can be very sensitive to them, but their knowledge is often limited or incomplete.

Qualitative approaches are more suitable to model gene regulatory networks and decipher their biological complexity [112]. More economical in parameters, they gain in flexibility by allowing the variables to represent different biological realities (activation of a gene or phosphorylation of a protein for example). Logical models are therefore much more abstract and qualitative representations of biological systems than models based on systems of differential equations [113]. See Appendix A for a presentation of logical modeling. They have demonstrated their effectiveness in a variety of biological systems [114], such as the regulation of the cellular response to DNA damage [115] or the combinatorial effect of mutations in tumorigenesis [116]. The emergence of next generation sequencing and the amount of data generated have facilitated the study of molecular regulatory networks helping the construction of numerous logical models, among others related to hematopoiesis. These models have been useful in understanding regulatory events in hematopoiesis, such as those focused on T cell differentiation [117,118] and their activation [119], which have contributed to the understanding of cancer resistance to immunotherapies [120].

Logical models have been particularly helpful to describe the dynamical behavior and differentiation of the progenitor and stem cell compartment. A logical model of differentiation starting from the CMP toward erythrocytic, megakaryocytic, granulocytic and monocytic lineages recapitulated the differentiation hierarchy characterized by the presence of an initial branching between granulocyte-monocyte progenitor (GMP) and megakaryocytes-erythrocyte progenitor (MEP) [121]. Further upstream in hematopoiesis, a logical model of HSPC regulation was established, presenting a stability in the dynamics that reflects the heterogeneity of the hematopoietic stem and progenitor cell (HSPC) population at different stages of activation, observed in single cell expression data [122]. The analysis of the model showed that some transient external activations allow the modelled system to escape from this stability zone and reach a differentiated state (for example, the activation of GATA1 allowed system to reach an erythroid state). Another model described a differentiation pathway starting from MPP states and reaching two stable states, corresponding to lymphoid and myeloid progenitors [123]. Interestingly, stimulations of the model with cytokines suggested a possible reprogramming of pre-B cells into macrophages by transient activation of CEBPA [123]. These studies demonstrated that in addition to providing a global mechanistic vision of the observed phenomena (progenitor differentiation), the construction and analysis of logic models also allow the prediction of new local regulations and additional mechanisms.

Logical modeling has also been very useful to understand the effect of extrinsic signals on HSC behavior. This can involve modeling the interaction between two types of cells. For example, a logical model of the dialogue between HSCs and mesenchymal stromal cells (MSCs) revealed attractors representing states of the system where HSC and MSC are attached and detached, respectively. The model highlighted the role of aberrant NF-kB expression in the creation of a tumor microenvironment [124]. Another recent model of a molecular regulatory network governing HSC quiescence and activation has been elaborated [125]. In the model, synchronous simulations provided stable LTHSC, STHSC, and proliferating HSC states, depending on a combination of niche signals that promoted quiescence or cell cycle activation. This model uncovered a novel regulatory mechanism of p53 in homeostasis, involving ROS- and RAS-activated TF regulators [125]. Such qualitative analysis of the dynamics opens up interesting avenues for studying age-related extrinsic deregulations of HSCs and the key players that control the resulting changes.

## 7. Single Cell Data and Boolean Networks Inference to Understand HSC Aging

**Figure 4:**
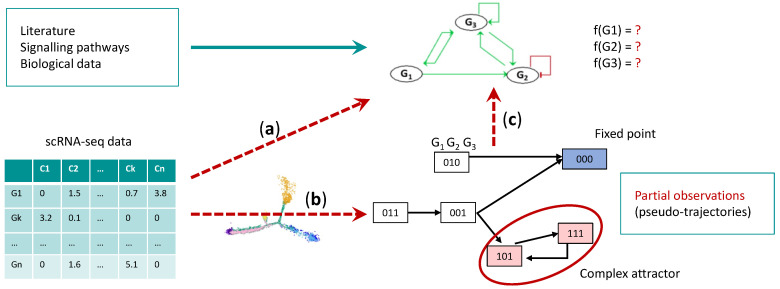
Boolean Network inference. Workflow of Boolean network inference from scRNA-seq data. (**a**) scRNA-seq data can be used to infer transcriptional interactions between TFs and complement influence graph constructed from prior knowledge of the searched BN. (**b**) The pseudo trajectories issued from the scRNA-seq data, can be discretized and translated in discrete observations of the searched BN. (**c**) Given these inputs, constraint programming can be used to infer the logical rules of the BN.

One of the current challenges is to adapt mathematical methods to single cell data in order to integrate as much information as possible extracted from the data into the Boolean model. This relies on inference methods, which have a two-fold purpose: the construction of the influence graph and the characterization of the Boolean parameters (Figure 4). This involves reverse engineering approaches.

### 7.1. Gene Regulatory Network Inference

The issue of inferring the molecular interaction network underlying the biological process has been addressed in the past from bulk expression data [126]. Nowadays, the amount of information provided by single-cell data has greatly improved the quality of inferring interactions between biological components of these networks, most notably for regulations between TFs and their target genes based on expression dependencies observed in the data. Numerous mathematical methods have been proposed for this purpose [127] and evaluated [128], relying on regression approaches [129,130], expression correlations [131] and information theory [132,133]. These approaches can be used to infer TF influence graph from single cell data and complement prior knowledge of gene regulatory network of the modelled biological system (Figure 4a). Inference of influence graphs from scRNA-seq data is clearly improved by adding additional levels of information, such as epigenetics and genomics. Epigenetic data from ChIP-seq experiments of TFs and histone marks, as well as ATAC-seq, have identified regulatory regions with DNA binding motifs for hundreds of TFs. These motifs were used to identify sets of genes coregulated by the same TF [134]. This strategy has been developed by an application, SCENIC, which searches for motif enrichments in the regulatory regions of a TF’s target genes by regression trees [60]. The combination of chromatin landscape, transcriptome, and other omics analyses at the single-cell level promises new developments in methods for inferring contextualized and accurate influence graphs of transcriptional interactions [127]. For example, scATAC-seq data have been used to link DNA regulatory elements to their potential target genes to reconstruct TF networks [135].

### 7.2. Boolean Network Inference

Mathematical models present the specificity of integrating the dynamics with the influence networks, thus enabling a better understanding of how the network of regulations gives rise to the global observed behavior. Model inference, therefore, requires the determination of the dynamical parameters, consistent with the influence graph. In the case of Boolean networks (BNs), this consists in inferring, for each node of the influence graph, a logical rule describing the changes of its state according to the state of its regulators. The trajectories of the global system will be defined from this set of logical rules. The pseudo-trajectories constructed from the scRNA-seq data can be interpreted as observations of some trajectories generated by a BN (Figure 4b). The challenge is then to find the logical rules from these “partial dynamics” that are sets of transitions between states observed in the data (Figure 4c). Several methods have been proposed to address this inference problem thanks to constraint programming, model checking and the introduction of novel semantics [34,136,137,138]. In practice, BN inference from scRNA-seq data have been successfully implemented to define a BN of embryonic hematopoietic development in humans [34], as well as a BN of HSC differentiation into MPP and MEP configurations [138].

Incorporation of new information from scRNA-seq into BN inference has necessitated, and thus enabled, the emergence of new analysis methods that are proving interesting and effective in capturing physiological changes, such as aging. Hence, Boolean models of aging have started to emerge. Schwab et al. [139] proposed an original analysis of single cell data exploiting population heterogeneity to sample time series data, from which sets of Boolean models were inferred. Then, by studying some topological properties, their analysis captured the dynamical heterogeneity, occurring with HSCs aging. Hérault et al. [140] presented an original inference methodology to construct a Boolean model allowing a better understanding of the mechanisms and factors controlling the effects of aging on HSCs. They developed an inference strategy that consists in a specific combination of different methods ranging from transcriptional regulation analysis for influence graph inference to constraint programming [136] and model checking techniques for logical rule inference. They succeeded in obtaining a Boolean model which agrees with most prior experimental observations of HSC biology. They provided expected and new predictions that could explain the myeloid bias in aged HSC differentiation, including the overactivation of Egr1 and Junb or/and the loss of Cebpa activation by Gata2 [140].

## 8. Conclusions

One of the limitations in understanding the mechanisms responsible for HSC aging is the heterogeneity of the cells, whose evolution plays a crucial role in age-related clonal changes. Single cell technologies are powerful tools to grasp the molecular features of a cell population and to follow how these features evolve upon aging. Since aging is associated with increased risks to develop blood malignant disorders, deciphering the transcriptional networks involved in HSC aging at the single cell level is crucial to prevent disease development. Beyond physiological aging, the knowledge of these molecular mechanisms will also be beneficial for a more accurate patient stratification and for the design of innovative treatments targeting specific pathways/mutations (“precision medicine”). One of the current challenges is to adapt mathematical based methods to single cell data and develop the corresponding tools to be able to understand the biological process under the study. In this line, computational models anchored in biology are ushering in a new era of HSC biology and provide a better understanding of the interconnected perturbations that drive stem cell diseases.

## Figures and Tables

**Figure A1:**
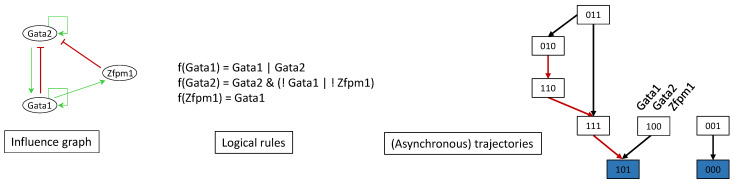
Example of Boolean network modeling for the three transcription factors Gata1, Gata2 and Zfpm1 in HSC erythroid-priming context. The influence graph represents the gene regulatory network whose dynamics is encoded with logical rules. Transitions between Boolean states (representative of cells at different times of differentiation) can be analyzed by asynchronously updating the components according to logical rules.

## Data Availability

Not applicable.

## Abbreviations

ARCH	Age-related clonal hematopoiesis
ATAC-seq	Assay for transposase-accessible chromatin with high throughput sequencing
BM	Bone marrow
BN	Boolean network
CCND2	Cyclin D2
CDC42	Cell division cycle 42
CDK6	*Cyclin-dependent kinase 6*
CDKN1A	Cyclin dependent kinase inhibitor 1A
CEBPA	CCAAT/enhancer-binding protein alpha
ChIP-seq	Chromatin immunoprecipitation followed by sequencing
CITE-seq	Cellular indexing of transcriptomes and epitopes by sequencing
CMP	Common myeloid progenitor
CRISPR-seq	Clustered regularly interspaced short palindromic repeats sequencing
DEG	Differentially expressed genes
DNA	Deoxyribonucleic acid
Drop-seq	Droplet-sequencing
EGR1	Early growth response protein 1
FACS	Fluorescence-activated cell sorting
GMP	Granulocyte-monocyte progenitor
HES1	Hairy and enhancer of split-1
HSC	Hematopoietic stem cell
HSPC	Hematopoietic stem and progenitor cell
HVG	Highly variable genes
JAK	Janus kinase
KLF	Krüppel-like factor
LTHSC	Long-term hematopoietic stem cell
MEP	Megakaryocytes-erythrocyte progenitor
MPP	Multipotent progenitor
MSC	Mesenchymal stromal cells
NF-kB	Nuclear factor kappa B
PCA	Principal component analysis
ROS	Reactive oxyygen species
RNA	Ribonucleic acid
scRNA-seq	single-cell RNA sequencing
STAT	Signal transducers and activators of transcription
STHSC	Short-term hematopoietic stem cell
TF	Transcription factor
TGF-β	Transforming Growth Factor Beta
t-SNE	t-distributed stochastic neighbor embedding
*UMAP*	*Uniform Manifold Approximation and Projection*

## Appendix A. Boolean Network Modeling Toolbox

An **influence graph** represents the regulations (activations or inhibitions) between selected biological components. Each component of the model is associated with a discrete variable standing for its activity level and logical parameters (using AND, OR and NOT operators) expressing the combined impact of its regulators. The global state of the system is represented by a discrete vector containing the activity values for all components.

For instance, in the example, we know that Gata2 is synergistically inhibited by Gata1 and Zfpm1 and that it activates its own promoter. Its **logical rule** is, therefore, Gata2 & (! Gata1|! Zfpm1). This rule indicates that Gata2 is ON if it is already present (self-activation) and at least one of the inhibitors is absent.

While the logical rules dictate the dynamics, the transitions between states are defined by the chosen semantic (updating rules) and determine the trajectories. With the **asynchronous dynamics**, whenever multiple components are called for a change, all single value changes are considered, leading to different possible trajectories from a same state. This dynamic is widely used in applications because it allows a state to have several successors, as can be observed in biology. Attractors in models are particularly studied because they represent the long-term behavior of systems. We distinguish two types of attractors, steady states (fixed points) or complex attractors (i.e., a set of nodes with oscillations). The biological analysis of the attractors from the levels of the states that constitute it allows to interpret and validate the model. In our example, if the system is in state 010 where only Gata2 is active (representing an uncommitted progenitor phenotype), it will go to the stable state 101 with Gata1 activated (primed erythrocyte phenotype).

The Boolean formalism enables the delineation of testable predictions by the simulations of mutants (KO or over expressed). These in silico perturbations allow us to simulate alterations in the system and then validate the model with genetic perturbation experiments and make predictions, all with minimal computational effort. Mutant simulations are performed by constraining the variables associated with the nodes, held at 0 for loss of function (KO mutant) or 1 for overexpression (KI mutants).
